# Identification of DNA methylation biomarkers from Infinium arrays

**DOI:** 10.3389/fgene.2012.00161

**Published:** 2012-08-25

**Authors:** Frank Wessely, Richard D. Emes

**Affiliations:** School of Veterinary Medicine and Science, University of NottinghamLoughborough, UK

**Keywords:** DNA methylation array, Infinium 450k, biomarker discovery, differential methylation, epigenetics, DNA methylome, epigenomics, NIMBL

## Abstract

Epigenetic modifications of DNA, such as cytosine methylation are differentially abundant in diseases such as cancer. A goal for clinical research is finding sites that are differentially methylated between groups of samples to act as potential biomarkers for disease outcome. However, clinical samples are often limited in availability, represent a heterogeneous collection of cells or are of uncertain clinical class. Array-based methods for identification of methylation provide a cost-effective method to survey a proportion of the methylome at single base resolution. The Illumina Infinium array has become a popular and reliable high throughput method in this field and are proving useful in the identification of biomarkers for disease. Here, we compare a commonly used statistical test with a new intuitive and flexible computational approach to quickly detect differentially methylated sites. The method rapidly identifies and ranks candidate lists with greatest inter-group variability whilst controlling for intra-group variability. Intuitive and biologically relevant filters can be imposed to quickly identify sites and genes of interest.

## Introduction

Studying DNA methylation profiles and related implications in developmental processes and diseases is currently the focus of much epigenetic research e.g., (Teschendorff et al., 2010; Sproul et al., 2011). Recent advances in the field of DNA methylation are due to a large range of experimental methods to detect genome-wide DNA methylation patterns. Amongst these, DNA methylation arrays, in particular the Illumina Infinium Human-Methylation BeadChips (Bibikova et al., 2011; Sandoval et al., 2011) provide a cost-effective platform to quantitatively measure methylation levels at single-base resolution. Their quantitative accuracy in comparison to other methods has been shown in recent studies (Bibikova et al., 2011; Dedeurwaerder et al., 2011; Touleimat and Tost, 2012). This is particularly clear when differences between compared groups are substantial, as non-uniform variation in the mid-range (heteroscedasticity) may mask true differences (Roessler et al., 2012).

When using these arrays, a common goal is to find CpG sites that are differentially methylated between two groups of samples, for example, in order to identify diagnostic biomarkers for diseases like cancer. Statistical tests, like the non-parametric Wilcoxon rank sum test, present an obvious approach to detect differential methylation between groups and are used in tools developed to analyse methylation arrays. These approaches may be combined with the ability to rank statistically significant sites according to the absolute difference between the average methylation levels of the analysed groups e.g., (Kanduri et al., 2010; Øster et al., 2011). However, in clinical cases, these approaches are often limited by the rarity of material, resulting in groups of limited numbers and an underpowered tool. Also, if a filtering step of absolute differences is not applied, retrieved candidates can represent statistically significant candidates which may be biologically irrelevant or difficult to validate by other means such as pyrosequencing (Roessler et al., 2012). Moreover, potential sites can be difficult to detect, if the sample DNA is obtained from a heterogeneous population of cells.

Here, we present Numerical Identification of Methylation Biomarker Lists (NIMBL), an approach to identify potential candidates, which uses a simple heuristic to identify and visualize differential methylation patterns between groups of samples. Importantly, NIMBL can also be used to compare and visualize the results obtained by multiple Infinium array investigation tools.

## Results

### Identification of differentially methylated sites using NIMBL

The input to NIMBL consists of a matrix of unique identifiers of each measured site and the paired measurements obtained for each sample: an unmethylated and methylated average hybridization signal, along with a detection *p*-value serving as a measure of probe performance. Additionally, annotation is provided for all array sites, setting them in genomic context by specifying chromosomal location, association with a gene or with a CpG island, etc. Methylation levels are usually estimated by calculating the proportion of the methylated signal to the sum of both signals and a constant offset, which avoids an overestimation if measured signals are low (Bibikova et al., 2009). These estimates are called beta values and can easily be interpreted as the proportion of methylation at a given locus. Thus, beta values range from 0 (CpG site always found unmethylated in sample DNA) to 1 (CpG site always found methylated in sample DNA). If beta values are already provided as part of the input, these values are used for analysis.

NIMBL consists of four main modules. The first module (NIMBL-qc) allows for a basic quality assessment of samples. Several output plots are generated to visualize the sample quality including a plot of the beta value distribution of each sample. Deviation from the expected distribution is largely related to the detection *p*-values, where an increase in number of measurements with low confidence of methylation accuracy (e.g., detection *p*-value >0.05) is reflected by a significant deviation of beta value distribution. A Kolmogorov–Smirnov test is performed to assist the identification of low quality samples which may influence downstream analysis and these can be then excluded from further analysis.

**Figure 1:**
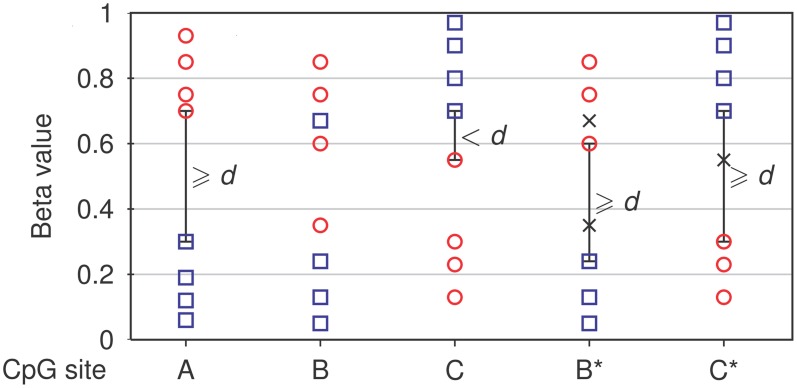
**Detection of differential methylation by NIMBL.** Each site is tested for a minimum distance of beta values (*d*) between two groups (corresponding samples depicted as circles and squares). If no samples are masked (*m* = 0), only non-overlapping sites separated by at least a value of *d* are identified **(A)**. If samples are masked (marked as x), sites with previously overlapping groups **(B)** or low discrimination **(C)** are additionally identified and flagged (B^*^,C^*^).

The core module of NIMBL is used to identify differentially methylated sites. Detection of differential methylation requires the selection of any two groups of samples, for example, tumor samples and controls. Array sites, where measurements are missing or show low confidence in a number of samples, can be excluded from differential methylation analysis by user-defined thresholds. Optionally, specific groups of sites can be selected using their annotation, for example, to include only autosomes in the analysis or restrict analysis to sites within CpG islands. Differentially methylated sites are identified as sites with the largest difference in methylation levels between the two groups. The user can control this by specifying a minimum beta value distance (*d*) between non-overlapping groups (Figure 1).

NIMBL has been developed in collaboration with researchers working with clinical data particularly those derived from tumor samples. These data have peculiarities which have guided some of the features of NIMBL. One of which is a method to cope with higher heterogeneous methylation common in primary tissue samples, particularly tumor samples when compared to cell lines [as has been noted previously (Bibikova et al., 2011; Roessler et al., 2012)]. This problem is increased in surgically removed samples which often include histopathologically normal cells resulting from the resection margin surrounding a removed tumor. To this end we have incorporated a user option to mask a proportion of samples which are highly heterogeneous. This option may also be useful to identify biomarkers where a sample cannot confidently be placed into a clinical group. If appropriate, the user specifies a maximum fraction of samples (*m*), which can be masked from each group (Figure 1, see Materials and Methods). Finally, the list of potential biomarkers is ranked by calculating a score based on the inter-group and intra-group variability:

(1) $$\text{score}=\text{beta}\_{\text{val}}_{\text{dist}}-({\text{median}}_{\text{diff}}-\text{beta}\_{\text{val}}_{\text{dist}})$$

where beta_val_dist_ is the distance in beta values between non-overlapping groups and median_diff_ is the absolute difference of the medians of each group. Higher discrimination between groups and lower variability within groups yields a higher score. The ranked list of potential biomarkers is visualized in a summary plot and reported along with annotation information in a text file. Besides the default procedure, further constraints can be imposed to test a biological question. These constraints can, for example, impose biologically motivated limits on methylation levels within one group, e.g., hypomethylated in a single group by limiting the beta value range allowed for that group. Additionally, the core module generates a comprehensive table of genes associated with the sites identified. This gene-centric analysis allows the rapid investigation of enrichment of differentially methylated sites within the genes and their corresponding regions (e.g., promoter regions).

The third module (NIMBL-gene) allows the detailed examination of user defined genes of interest. Genomic information is used to create methylation overview plots of each gene, where methylation measurements are plotted according to their genomic location. Moreover, alignment files in the FASTA format, which contain the DNA sequence of each gene and the aligned DNA probe sequences, provide information of the exact sequence context of array measurements.

Finally, the fourth module (NIMBL-compare) can be used to compare two or three lists of sites identified by any method or by different settings of one method. The comparison is performed both on a site level and gene level. The gene information tables, similar to the one obtained from the core module, can be used to identify candidate genes that are common or unique between the input lists or their subsets.

### Comparison of methods to detect differentially methylated sites

Whilst no universally accepted method for the analysis of Infinium arrays exists; widely used approaches are incorporated within the Illumina Methylation Analyzer (IMA) (Wang et al., 2012). Whilst IMA has many variables, common usage is to conduct the Wilcoxon rank-sum test for inference of differences between categorical groups and a general linear model (using the limma R package) to infer methylation change associated with a continuous covariate (Smyth, 2005).

A publicly available breast cancer data set consisting of eight tumor and eight normal samples (Dedeurwaerder et al., 2011) was used as the test data (see Materials and Methods). IMA was run using both Wilcoxon rank sum test and linear model (limma) approaches and the results obtained were compared. The NIMBL-compare module was used to visualize overlap and uniquely identified differentially methylated sites.

**Figure 2:**
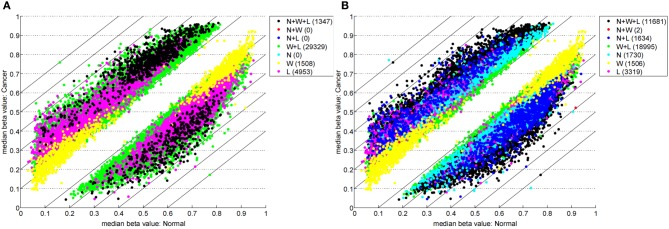
**Comparison of methylation levels without post-filter.** NIMBL-compare output of median beta values of normal versus cancer samples when no samples (*m* = 0) **(A)** or up to two samples (*m* = 2) **(B)** were masked by NIMBL. The number of sites unique to each of the seven sets are given in brackets. Abbreviations: N, NIMBL; W, IMA wilcoxon; L, IMA limma.

**Figure 3:**
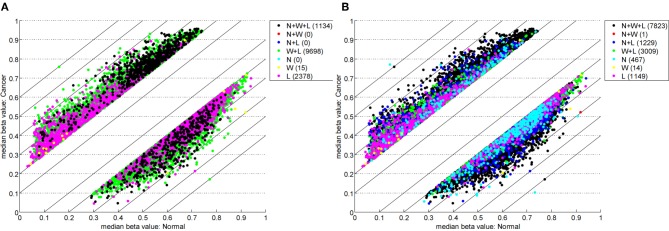
**Comparison of methylation levels with post-filter.** NIMBL-compare output of median beta values of normal versus cancer samples when no samples (*m* = 0) **(A)** or up to two samples (*m* = 2) **(B)** were masked by NIMBL and sites were selected by a minimum median beta value difference of 0.2. The number of sites unique to each of the seven sets are given in brackets. Abbreviations: N, NIMBL; W, IMA wilcoxon; L, IMA limma.

Table 1 highlights that when no minimum median difference between groups is imposed (filter = no) many (11,681) sites are identified by all three methods representing approximately 78% of those detected by NIMBL [*d* = 0.1, *m* = 2]. Substantial differences between the three approaches are seen with 1730, 1506, and 3319 sites identified by only a single method (NIMBL [*d* = 0.1, *m* = 2], Wilcoxon and limma, respectively). The scatterplot output of NIMBL-compare shows that whilst the value of *m* influences overlap (Table 1, Figure 2), the majority of sites not identified by NIMBL are due to small differences between groups. When a minimum beta value median difference between groups of 0.2 is imposed (Table 1, filter = yes), the number of sites identified by all methods reduces by approximately 33% (7823 sites identified by all methods). The number of sites identified by an individual method also substantially reduces (467 NIMBL [*d* = 0.1, *m* = 2], 14 Wilcoxon and 1149 limma, see Table 1 and Figure 3). Whilst imposition of a minimum median beta value difference of 0.2 may appear arbitrary, this is a cut-off that has been shown to allow robust confirmation of findings by alternative methods such as sequencing of sodium bisulfite converted DNA, which is considered the gold standard method for quantifying DNA methylation (Emes and Farrell, 2012).

**Table 1:** **Number of differentially methylated sites**.

***d***	***m***	**Filter**	**N**_**tot**_	**W**_**tot**_	**L**_**tot**_	**N+W+L**	**N+W**	**N+L**	**W+L**	**N**	**W**	**L**
0.1	0	No	1347	32,184	35,629	1347	0	0	29,329	0	1508	4953
0.1	1	No	5740	32,184	35,629	5351	0	318	25,325	71	1508	4635
0.1	2	No	15,047	32,184	35,629	11,681	2	1634	18,995	1730	1506	3319
0.1	0	Yes	1134	10,847	13,210	1134	0	0	9698	0	15	2378
0.1	1	Yes	4457	10,847	13,210	4186	0	256	6646	15	15	2122
0.1	2	Yes	9520	10,847	13,210	7823	1	1229	3009	467	14	1149

Sites identified by each method were compared against each other. Overlapping and unique sites are reported. Abbreviations: *d*, minimum inter-group distance within NIMBL; *m*, maximum number of masked samples in each group within NIMBL; filter, minimum median beta value difference of 0.2 between groups; N, NIMBL; W, IMA Wilcoxon test; L, IMA limma test; tot, total number of sites identified by each method.

This comparison highlights the lack of concordance between NIMBL and IMA and also between the limma and Wilcoxon methods of IMA. Importantly due to the variability of methylation between individuals and cells, reporting of single sites which are differentially methylated is unlikely to identify epigenetic modifications relating to changes in phenotype. To achieve this, a gene- or region-centric approach should be undertaken. One such approach known as the “bump hunting” method (Jaffe et al., 2012), which was originally developed for CHARM arrays (Irizarry et al., 2008), is also applicable to Infinium methylation array data. However, the lower density of measurements of the Infinium compared to CHARM arrays leads to a significant subset of unused measurements (Jaffe et al., 2012). An alternative is to collect and summarize methylation measurements for specific gene regions (e.g., proximal to transcriptional start sites) as is conducted by IMA. This has been suggested to improve statistical power by conducting fewer individual tests (Wang et al., 2012). To summarize data for a region, NIMBL reports the number of sites measured and the number of these detected as differentially methylated within different annotated regions of each gene. This approach allows for a rapid identification of genes and gene regions of interest.

When the numbers of sites from each approach are mapped to underlying genes an increase in overlap between methods is seen (Table 2). 4678 genes are identified as differentially methylated by all methods; this is approximately 89% of the 5282 differentially methylated genes detected by NIMBL (Table 2).

**Table 2:** **Number of differentially methylated genes**.

***d***	***m***	**Filter**	**N**_**tot**_	**W**_**tot**_	**L**_**tot**_	**N+W+L**	**N+W**	**N+L**	**W+L**	**N**	**W**	**L**
0.1	0	No	772	7871	8465	772	0	0	6978	0	121	715
0.1	1	No	2613	7871	8465	2550	0	46	5200	17	121	669
0.1	2	No	5282	7871	8465	4678	6	283	3072	315	115	432
0.1	0	Yes	665	3918	4532	665	0	0	3249	0	4	618
0.1	1	Yes	2074	3918	4532	1999	0	69	1915	6	4	549
0.1	2	Yes	3678	3918	4532	3207	0	341	707	130	4	277

Genes identified by each method were compared against each other. Overlapping and unique genes are reported. N, NIMBL; W, IMA Wilcoxon test; L, IMA limma test; tot, total number of genes identified by each method.

**Figure 4:**
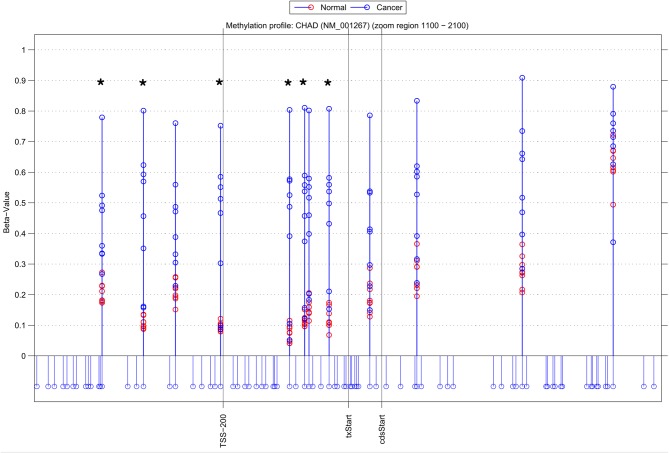
**Methylation profile of gene CHAD.** NIMBL-gene output showing a zoomed region of the genomic location of the gene CHAD displaying 12 out of 14 CpG sites associated with this gene on the array. The stems below the x-axis correspond to all CpG sites located in this region. CpG sites identified as differentially methylated by NIMBL [*d* = 0.1, *m* = 2] are highlighted with asterisks. Abbreviations: TSS200, position 200 bp upstream of transcriptional start site (txStart); cdsStart, coding sequence start.

Using NIMBL-gene a user defined list of candidate genes can be investigated in greater detail. Figure 4 shows an example graphical output of NIMBL for the gene chondroadherin (CHAD) identified as differentially methylated by all compared methods. In the test data the CHAD gene is clearly hypermethylated in the cancer samples. This increase in methylation is particularly distinct in the region in close proximity to the transcriptional start site (TSS-200 – txStart in Figure 4). Although this is a good candidate for an epigenetically regulated gene, this example also highlights that individual cancer samples can be particularly heterogeneous with one or two cancer samples having a methylation profile more similar to normal samples. It was the observation of these examples which drove the NIMBL approach to allow masking of a proportion of sites when identifying differentially methylated candidates.

**Figure 5:**
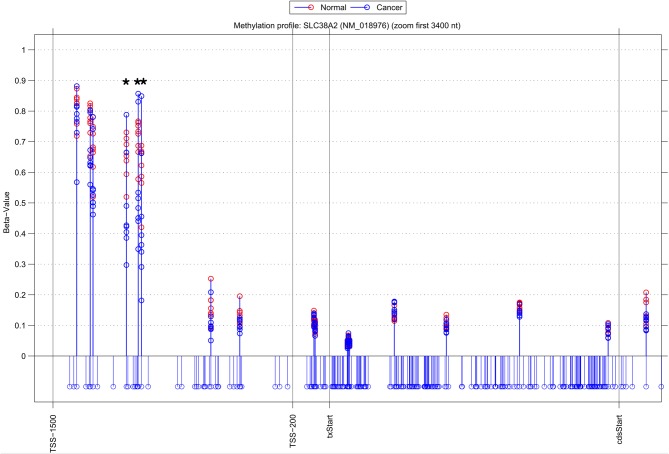
**Methylation profile of gene SLC38A2.** NIMBL-gene output showing a zoomed region of the genomic location of the gene SLC38A2 displaying 18 out of 20 CpG sites associated with this gene on the array. The stems below the x-axis correspond to all CpG sites located in this region. CpG sites identified as differentially methylated by NIMBL [*d* = 0.1, *m* = 2] are highlighted with asterisks. Abbreviations: TSS1500, position 1500 bp upstream of transcriptional start site; TSS200, position 200 bp upstream of transcriptional start site (txStart); cdsStart, coding sequence start.

Whilst there is general agreement between methods, a substantial number of genes are also identified by a single approach (315 NIMBL [*d* = 0.1, *m* = 2], 115 Wilcoxon and 432 limma, see Table 2). The number of genes identified by a single approach is again reduced when a minimum median difference between groups of 0.2 is imposed (130 NIMBL [*d* = 0.1, *m* = 2], 4 Wilcoxon and 227 limma, see Table 2). One of the 130 genes identified by NIMBL alone as being an epigenetically regulated candidate is solute carrier family 38, member 2 (SLC38A2). Three sites upstream of the transcriptional start site were identified by NIMBL as being hypomethylated in cancer samples (Figure 5). Generally hypomethylation of a promoter region will be associated with expression of the gene in question. In this case we would predict hypomethylation of the potential promoter region of SLC38A2 would result in higher expression of SLC38A2 in the breast cancer samples analyzed. Although this would require verification, SLC38A2 gene expression is found to be upregulated in various cell lines including ssMCF7 breast cancer and HMEC184 breast cancer (Lukk et al., 2010).

## Discussion

Recent emphasis on the unraveling of the epigenome has resulted in a number of methods for the identification of differential methylation and this continues to be an active area of research e.g., (Du et al., 2008; Barfield et al., 2012; Kilaru et al., 2012; Wang et al., 2012). From our interactions with clinical researchers, particularly those interested in comparison of disease with clinically normal samples, we identified that a biologically intuitive method for the interpretation and prioritization of candidate biomarkers would be useful to the research community. We have developed an intuitive numerical approach that can quickly test various biological hypotheses to retrieve a list of candidates, which can be subject for follow-up studies. Our method is focused on incorporating biologically meaningful constraints, thereby taking advantage of the easy interpretation of beta values. An alternative method to quantify methylation levels for the Infinium array (M-value) (Irizarry et al., 2008) has been suggested to outperform beta values in detection of differentially methylated CpG sites (Du et al., 2010) However, we use beta values as they are robust to the type of methylation quantification (Bell et al., 2011) and the values are easily interpretable in a biological context.

It is important to note that none of the available methods including NIMBL will be guaranteed to provide a list of true positives, but these methods are invaluable to prioritize genes of particular interest to the researcher. To aid with this interpretation we developed modules of NIMBL that allow comparisons of different methods, visualize differentially methylated sites in the context of the gene, and provide the genomic DNA and probe information so that confirmatory experiments can be quickly conducted.

The results obtained using the freely available test data showed that the majority of sites identified by NIMBL are also identified by two common approaches (Wilcoxon test and limma procedure), which are both available within IMA. Many sites were detected by IMA using the limma and/or Wilcoxon tests. However, many of these were due to small differences (median beta value difference <0.2) between groups (Table 1). Whilst some of these are potentially of interest, most will not be readily verifiable by sequencing methods.

When a minimum median beta value difference of 0.2 post-filter is used, many of these additional sites are removed, although a significant number (3023) remain undetected by NIMBL but detected by IMA with the Wilcoxon option. Wilcoxon is the default option of IMA for comparison of categorical data which we have in this test example and is therefore expected to be more appropriate than the limma method. However, there is significant overlap between the Wilcoxon and limma approaches of IMA with the limma option producing the largest number of candidate sites. Limma is an R package for linear modeling of experimental data and was developed for use in the analysis of microarray data (Smyth, 2005). As the experiment conducted here used categorical data (normal vs. cancer data) it is possible that the use of limma is inappropriate and has identified some false positives and potentially only those also identified using the appropriate Wilcoxon method should be considered.

To overcome the problems when comparing clinical samples such as tumor samples with high heterogeneity, the potential of mis-diagnosis or analysis of a mixed population of cells, NIMBL was developed to allow the masking of a proportion of values from each compared group. The effect of the masking variable can be seen in Table 1 and Figure 2. When *m* = 1 a total of 5740 sites are identified as differentially methylated by NIMBL. 5669 (98.8%) of these are also identified by IMA using either the limma or Wilcoxon method. As the number of masked sites increases the number of NIMBL-specific sites also increases. For example, when *m* = 2 a total of 15,047 sites are identified as differentially methylated by NIMBL and the majority of these (13,317, 88.5%) are also identified by IMA using either the limma or Wilcoxon method whilst a small proportion (1730) of CpGs are detected by NIMBL alone. This may be seen as a controversial approach as the removal of highly variable sites would invalidate the principles of statistical testing. However, the number of individual samples masked is reported in the NIMBL output and should be used as one of the criteria in ranking candidates for verification. We are confident that this option offers flexibility for the user and this approach has identified a number of candidates of interest by ourselves, for example, the identification of SLC38A2 identified here as a potentially epigenetically regulated gene which is differentially expressed in breast cancer cell lines.

The quality control implemented within NIMBL allows a quick assessment of overall sample performance. An aberrant beta value distribution of samples detected by NIMBL is used as an indicator of a low quality sample on the array. However, such a deviation might be due to biological reasons (e.g., large chromosomal aberrations) rather than technical failure. The user is advised to integrate all quality control information, especially the number of low quality measurements of each sample. A correlation between these data and an aberrant beta value distribution is a strong indicator of an experimental problem. Other tools, for example, the R packages lumi (Du et al., 2008), methylumi or HumanMeth27QCReport (Mancuso et al., 2011), offer comprehensive quality control procedures and could be used if required.

Several technical factors have been identified to impact the methylation data obtained from Infinium arrays, including batch effects, color bias, and a methylation level shift due to two different probe designs (commonly referred to Infinim I and II) that are used for the latest array technology (Dedeurwaerder et al., 2011; Sun et al., 2011; Touleimat and Tost, 2012). The probe design compensation is considered an important correction by multiple groups. NIMBL offers the option to correct Infinium II signals as described in Dedeurwaerder et al. (2011). However, two alternative methods have also been proposed recently (Maksimovic et al., 2012; Touleimat and Tost, 2012). No absolute standard for data preprocessing has been established yet and further studies are necessary to evaluate and integrate different correction approaches within preprocessing pipelines as was shown recently in Touleimat and Tost (2012). Whilst the methods to preprocess samples may change, the output of preprocessed beta values from any preprocessing tools can easily serve as the input for NIMBL or multiple methods can be compared with NIMBL-compare.

NIMBL was developed as a methylation analysis pipeline which can be used by researchers in an easy and intuitive way without requiring detailed programming knowledge. Setting methylation measurements in their genomic context significantly facilitates the biological interpretation of methylation array data.

## Materials and methods

### Datasets

The methylation dataset used in this study was retrieved from the Gene Expression Omnibus (GEO) database (GEO accession GSE29290). This dataset consists of eight breast tumor samples and eight normal breast tissue samples (Dedeurwaerder et al., 2011), for which methylation levels were measured with the Illumina HumanMethylation450 BeadChip. The obtained data files include the methylation levels as beta values and corresponding detection *p*-values to estimate the quality of the measurement. The HumanMethylation450_15017482_v.1.1 manifest from Illumina was used for annotation of array sites (including, for example, gene and gene region information). Genomic information including genomic DNA sequence and coordinates of gene coding regions were obtained from the UCSC Genome Browser database (Dreszer et al., 2012).

### Software

Two software packages were used for methylation analysis. First, NIMBL (Numerical identification of Methylation Biomarker Lists) is a Matlab package which is freely available from: https://sites.google.com/site/emesbioinformatics/group-software. Second, IMA (Illumina Methylation Analyzer, version 3.1.2) (Wang et al., 2012) is an R package, which is freely available from: http://www.rforge.net/IMA/.

### Data pre-processing

Array sites with one or more of the 16 samples having a detection *p*-value of less than 0.05 and sites with missing measurements (beta values) were excluded from differential methylation analysis in both software packages. The final dataset contained 480,917 measurements as the input of both packages.

### Differential methylation analysis

The detection of differentially methylated sites by NIMBL is controlled by setting a minimum beta value distance (*d*) between non-overlapping groups. Optionally, a maximum number of samples can be masked within each group. Two different masking limits for each group can be specified within NIMBL. We used the same value (*m*) for each group within this study. Masking of samples is performed for each array site independently. Group medians are calculated and the distance of each sample to the corresponding group median is determined. The sample most distant to its group median is masked and remaining samples are re-tested for *d*. A single sample is masked per iteration irrespective of group. This process repeats until either *d* is reached or *m* samples are masked for each group. Here, the differential methylation settings used for NIMBL are based on a constant level of beta value inter-group distance (*d* = 0.1) and three different values for the maximum number of masked samples within each group (*m* = 0, 1 or 2). The NIMBL input script for the complete methylation analysis of the test data can be obtained from the example directory within the NIMBL package.

To detect differential methylation by IMA, the Wilcoxon and limma methods were applied. The Benjamini–Hochberg (BH) procedure was used for multiple testing correction of both IMA methods and the cut-off for adjusted *p*-values was set to 0.05. NIMBL-compare was used to impose a post-filter of a median methylation difference of 0.2 between the two groups.

### Conflict of interest statement

The authors declare that the research was conducted in the absence of any commercial or financial relationships that could be construed as a potential conflict of interest.

## References

[B1] Barfield R. T. Kilaru V. Smith A. K. Conneely K. N. (2012). CpGassoc: an R function for analysis of DNA methylation microarray data. Bioinformatics 28, 1280–1281 10.1093/bioinformatics/bts124 22451269 PMC3577110

[B2] Bell J. T. Pai A. A. Pickrell J. K. Gaffney D. J. Pique-Regi R. Degner J. F. Gilad Y. Pritchard J. K. (2011). DNA methylation patterns associate with genetic and gene expression variation in HapMap cell lines. Genome Biol. 12, R10 10.1186/gb-2011-12-1-r10 21251332 PMC3091299

[B3] Bibikova M. Barnes B. Tsan C. Ho V. Klotzle B. Le J. M. Delano D. Zhang L. Schroth G. P. Gunderson K. L. Fan J.-B. Shen R. (2011). High density DNA methylation array with single CpG site resolution. Genomics 98, 288–295 10.1016/j.ygeno.2011.07.007 21839163

[B4] Bibikova M. Le J. Barnes B. Saedinia-Melnyk S. Zhou L. Shen R. Gunderson K. L. (2009). Geome-wide DNA methylation profiling using Infinium assay. Epigenomics 1, 177–200 10.2217/epi.09.14 22122642

[B5] Dedeurwaerder S. Defrance M. Calonne E. Denis H. Sotiriou C. Fuks F. (2011). Evaluation of the Infinium Methylation 450K technology. Epigenomics 3, 771–784 10.2217/epi.11.105 22126295

[B6] Dreszer T. R. Karolchik D. Zweig A. S. Hinrichs A. S. Raney B. J. Kuhn R. M. Meyer L. R. Wong M. Sloan C. A. Rosenbloom K. R. Roe G. Rhead B. Pohl A. Malladi V. S. Li C. H. Learned K. Kirkup V. Hsu F. Harte R. A. Guruvadoo L. Goldman M. Giardine B. M. Fujita P. A. Diekhans M. Cline M. S. Clawson H. Barber G. P. Haussler D. James Kent W. (2012). The UCSC Genome Browser database: extensions and updates 2011. Nucleic Acids Res. 40(Database issue), D918–D923 10.1093/nar/gkr1055 22086951 PMC3245018

[B7] Du P. Kibbe W. A. Lin S. M. (2008). lumi: a pipeline for processing Illumina microarray. Bioinformatics 24, 1547–1548 10.1093/bioinformatics/btn224 18467348

[B8] Du P. Zhang X. Huang C.-C. Jafari N. Kibbe W. A. Hou L. Lin S. M. (2010). Comparison of Beta-value and M-value methods for quantifying methylation levels by microarray analysis. BMC Bioinformatics 11, 587 10.1186/1471-2105-11-587 21118553 PMC3012676

[B9] Emes R. D. Farrell W. E. (2012). Make way for the ‘next generation’: application and prospects for genome-wide, epigenome-specific technologies in endocrine research. J. Mol. Endocrinol. 49, R19–R27 10.1530/JME-12-0045 22525352

[B10] Irizarry R. A. Ladd-Acosta C. Carvalho B. Wu H. Brandenburg S. A. Jeddeloh J. A. Wen B. Feinberg A. P. (2008). Comprehensive high-throughput arrays for relative methylation (CHARM). Genome Res. 18, 780–790 10.1101/gr.7301508 18316654 PMC2336799

[B11] Jaffe A. E. Murakami P. Lee H. Leek J. T. Fallin M. D. Feinberg A. P. Irizarry R. A. (2012). Bump hunting to identify differentially methylated regions in epigenetic epidemiology studies. Int. J. Epidemiol. 41, 200–209 10.1093/ije/dyr238 22422453 PMC3304533

[B12] Kanduri M. Cahill N. Göransson H. Enström C. Ryan F. Isaksson A. Rosenquist R. (2010). Differential genome-wide array-based methylation profiles in prognostic subsets of chronic lymphocytic leukemia. Blood 115, 296–305 10.1182/blood-2009-07-232868 19897574

[B13] Kilaru V. Barfield R. T. Schroeder J. W. Smith A. K. Conneely K. N. (2012). MethLAB: a graphical user interface package for the analysis of array-based DNA methylation data. Epigenetics 7, 225–229 10.4161/epi.7.3.19284 22430798 PMC3335946

[B14] Lukk M. Kapushesky M. Nikkil J. Parkinson H. Goncalves A. Huber W. Ukkonen E. Brazma A. (2010). A global map of human gene expression. Nat. Biotechnol. 28, 322–324 10.1038/nbt0410-322 20379172 PMC2974261

[B15] Maksimovic J. Gordon L. Oshlack A. (2012). SWAN: Subset quantile Within-Array Normalization for Illumina Infinium HumanMethylation450 BeadChips. Genome Biol. 13, R44 10.1186/gb-2012-13-6-r44 22703947 PMC3446316

[B16] Mancuso F. M. Montfort M. Carreras A. Alibés A. Roma G. (2011). HumMeth27QCReport: an R package for quality control and primary analysis of Illumina Infinium methylation data. BMC Res. Notes 4, 546 10.1186/1756-0500-4-546 22182516 PMC3285701

[B17] Øster B. Thorsen K. Lamy P. Wojdacz T. K. Hansen L. L. Birkenkamp-Demtröder K. Sørensen K. D. Laurberg S. Ørntoft T. F. Andersen C. L. (2011). Identification and validation of highly frequent CpG island hypermethylation in colorectal adenomas and carcinomas. Int. J. Cancer 129, 2855–2866 10.1002/ijc.25951 21400501

[B18] Roessler J. Ammerpohl O. Gutwein J. Hasemeier B. Anwar S. L. Kreipe H. H. Lehmann U. (2012). Quantitative cross-validation and content analysis of the 450k DNA methylation array from Illumina, Inc. BMC Res. Notes 5, 210 10.1186/1756-0500-5-210 22546179 PMC3420245

[B19] Sandoval J. Heyn H. Moran S. Serra-Musach J. Pujana M. A. Bibikova M. Esteller M. (2011). Validation of a DNA methylation microarray for 450,000 CpG sites in the human genome. Epigenetics 6, 692–702 10.4161/epi.6.6.16196 21593595

[B20] Smyth G. K. (2005). Limma: linear models for microarray data, in Bioinformatics and Computational Biology Solutions using R and Bioconductor, eds Gentleman R. Carey V. Dudoit S. Irizarry R. Huber W. (New York, NY: Springer), 397–420

[B21] Sproul D. Nestor C. Culley J. Dickson J. H. Dixon J. M. Harrison D. J. Meehan R. R. Sims A. H. Ramsahoye B. H. (2011). Transcriptionally repressed genes become aberrantly methylated and distinguish tumors of different lineages in breast cancer. Proc. Natl. Acad. Sci. U.S.A. 108, 4364–4369 10.1073/pnas.1013224108 21368160 PMC3060255

[B22] Sun Z. Chai H. S. Wu Y. White W. M. Donkena K. V. Klein C. J. Garovic V. D. Therneau T. M. Kocher J.-P. A. (2011). Batch effect correction for genome-wide methylation data with Illumina Infinium platform. BMC Med. Genomics 4, 84 10.1186/1755-8794-4-84 22171553 PMC3265417

[B23] Teschendorff A. E. Menon U. Gentry-Maharaj A. Ramus S. J. Weisenberger D. J. Shen H. Campan M. Noushmehr H. Bell C. G. Maxwell A. P. Savage D. A. Mueller-Holzner E. Marth C. Kocjan G. Gayther S. A. Jones A. Beck S. Wagner W. Laird P. W. Jacobs I. J. Widschwendter M. (2010). Age-dependent DNA methylation of genes that are suppressed in stem cells is a hallmark of cancer. Genome Res. 20, 440–446 10.1101/gr.103606.109 20219944 PMC2847747

[B24] Touleimat N. Tost J. (2012). Complete pipeline for Infinium(®) Human Methylation 450K BeadChip data processing using subset quantile normalization for accurate DNA methylation estimation. Epigenomics 4, 325–341 10.2217/epi.12.21 22690668

[B25] Wang D. Yan L. Hu Q. Sucheston L. E. Higgins M. J. Ambrosone C. B. Johnson C. S. Smiraglia D. J. Liu S. (2012). IMA: an R package for high-throughput analysis of Illumina's 450K Infinium methylation data. Bioinformatics 28, 729–730 10.1093/bioinformatics/bts013 22253290 PMC3289916

